# Impact of Vaccination and Control Measures on the Fatality of COVID-19: An Ecological Study

**DOI:** 10.1007/s44197-022-00064-2

**Published:** 2022-09-26

**Authors:** Jinlin Tan, Zhilong Wu, Lin Gan, Qianhong Zhong, Yajuan Zhu, Yufen Li, Dingmei Zhang

**Affiliations:** 1 Department of Epidemiology, School of Public Health, Sun Yat-Sen University, Guangzhou, 510080 Guangdong China; 2 Department of Tuberculosis Control, The Fourth People’s Hospital of Foshan City, Foshan, 528000 Guangdong China

**Keywords:** COVID-19, Case fatality rate, Variant, Vaccination, Prevention and control measures

## Abstract

**Background:**

During the COVID-19 pandemic, reducing the case fatality rate (CFR) becomes an urgent goal.

**Objective:**

This study explored the effect of vaccination and variants on COVID-19 fatality and provide a basis for the adjustment of control measures.

**Methods:**

This study collected epidemiological information on COVID-19 from January to October 2021. By setting different lag times, we calculated the adjusted CFR. The Spearman correlation coefficient and beta regression were used to explore factors that may affect COVID-19 fatality.

**Results:**

Every 1% increase in the percentage of full vaccinations may reduce the 3 weeks lagging CFR by 0.66%. Increasing the restrictions on internal movement from level 0 to 1, restrictions on international travel controls from level 2 to 3, and stay-at-home restrictions from level 0 to 2 were associated with an average reduction in 3 weeks lagging CFR of 0.20%, 0.39%, and 0.36%, respectively. Increasing strictness in canceling public events from level 0 to 1 and 2 may reduce the 3 weeks lagging CFR by 0.49% and 0.37, respectively. Increasing the severity of school and workplace closures from level 1 or level 0 to 3 may increase the 3 weeks lagging CFR of 0.39% and 0.83, respectively. Every 1-point increase in the Global Health Security (GHS) index score may increase the 3 weeks lagging CFR by 0.12%.

**Conclusion:**

A higher percentage of full vaccinations, higher levels of internal movement restrictions, international travel control restrictions, cancelations of public events, and stay-at-home restrictions are factors that may reduce the adjusted CFR.

## Introduction

Following the outbreak of respiratory illness in 2019, the World Health Organization (WHO) announced that coronavirus disease 2019 (COVID-19) can be characterized as a pandemic on March 11, 2020 after assessment, and the pathogen was named as severe acute respiratory syndrome coronavirus-2 (SARS-CoV-2). The cases of COVID-19 range from asymptomatic to severe, and the most common symptoms are fever, cough, and fatigue [1, 2].

The fatality rate of COVID-19 differs with time. As of 2 September 2022, there were a total of 601,189,435 confirmed cases worldwide and a total of 6,475,346 deaths [3]. The crude case fatality rate (CFR) was 1.08%, which was lower than 4.14% on 17 March 2020 [4]. In addition to individual characteristics such as age, gender, and comorbidities [5], many factors at the national level, such as the public health system and poverty level, can also affect clinical outcomes [6]. Exploring the factors that affect lethality can provide a basis for adjusting targeted prevention and treatment measures.

Since it was first identified, SARS-CoV-2 has continued to evolve, which has attracted widespread attention from researchers, and the effects of virus mutation are still being studied. The WHO has named five variants of concern (VOCs) on the basis of gene similarity, namely, Alpha, Beta, Gamma, Delta, and Omicron [7]. Many studies have shown that these variants can lead to more serious disease outcomes and even higher fatality in infected individuals. The results of a cohort study conducted in the UK showed that Alpha VOC caused higher fatality compared with non-Alpha variants [8]. According to a study that compared the first wave of COVID-19 in South Africa with the second wave, the hospital fatality rate increased in the second wave, and part of the increase was attributed to Beta variants [9]. Shi Zhao et al. found that Gamma variants lead to a higher risk of death [10]. In a study from Denmark, patients infected with the Delta variant had a higher risk of hospitalization, but most of these cases were unvaccinated people [11]. Similarly, a Canadian study found that compared with non-VOCs, VOCs increase the risk of hospitalization and death [12].

Since 2020, pharmaceutical industries and governments have carried out unprecedented cooperation, leading to rapid progress in the development of COVID-19 vaccines [13–15]. Many countries have successively approved widespread vaccination against COVID-19. Many studies have shown that vaccines can reduce the risk of severe symptoms, hospitalization, and even death in infected people [16, 17]. Of note, vaccine breakthrough infections exist and are closely related to VOCs [18, 19]. Therefore, when analyzing the relationship between variants and fatality, we must consider the impact of vaccination.

Meanwhile, the treatment of COVID-19 has continuously improved. In addition, the measures taken by governments in response to the outbreak are continuously being adjusted. A study found that the formulation of various policies may affect the fatality rate [20]. To allow policymakers and citizens to understand the government’s policy response to COVID-19 in a consistent manner, the Oxford Covid-19 Government Response Tracker (OxCGRT) has collected information on the policy measures taken by governments from January 1, 2020, and compiled a set of policy indicators [21], including policies on school and workplace closures, cancelation of public events and gatherings, stay-at-home restrictions, and international and domestic travel. This provided a benchmark for comparison of policies in different countries. Advances in treatment methods and strictness of prevention and control policies may also affect the fatality rate of COVID-19. Consequently, this study explored the impact of various policy factors on the fatality rate of COVID-19.

However, due to the different pressures on the medical system, vaccination coverage rates, and treatment methods, the fatality rates of different VOCs are not comparable at different time points of the epidemic. On that account, multiple factors need to be considered comprehensively to compare the effects of variants on the fatality rate.

Therefore, when these VOCs exist at the same time, taking the proportion of each VOC as one of the independent variables and considering the existence of multiple VOCs simultaneously to explore the influencing factors of the fatality rate can make the results more convincing. To fully explore the factors that affect the fatality rate, this study also considered dependent variables such as the proportion of people who are fully vaccinated, life expectancy at birth that reflects the overall mortality level of the population, total health expenditure per capita that reflects the overall health expenditure level of the country, and various policy indicators.

Moreover, the most common method for estimating the CFR is to divide the number of reported deaths by the total number of cases reported at the same time. However, this method is too simple to take into account the delay between onset and death, which will result in an underestimation of the fatality rate [22]. Earlier studies pointed out that the median time from onset to death of COVID-19 patients was 18.5 days (15.0–22.0) [23]. A study of severe COVID-19 patients found that the median time from admission to the ICU to death was 15 days [24]. Consequently, this study adjusted the result delay in the CFR estimation and used ecological research to analyze the relationship between the prevalence of different variants and the CFR. To improve sensitivity, different delay times were adjusted.

This study adopted the design of ecological research, considered the changes in government policies at different time points, the proportion of vaccination, and other variables that cannot be specifically reflected in population studies; and explored the factors related to the fatality rate of the population from a more macro-level.

## Method

### Data Collection

The ISO week date system was used in this study for timekeeping, which is part of the ISO 8601 date and time standard published by the International Organization for Standardization (ISO) [25]. Weeks start on Monday and end on Sunday. The first week of each year is the week that contains Thursday. The first week of 2021 started on January 4.

Four countries, namely, UK, Denmark, Switzerland, and Norway, were included in this study. According to the WHO COVID-19 detailed surveillance data dashboard [26], this study obtained information about confirmed COVID-19 cases and COVID-19 death cases every week from January 4, 2021, to October 24, 2021.

Developed by the UK Health Security Agency, the official website of the British government provides a proportion of SARS-CoV-2 VOCs in the UK [27]. The Norwegian Institute of Public Health publishes weekly reports for coronavirus and COVID-19 [28], which is the source of the proportion of VOCs in Norway in this study. The Swiss Federal Office of Public Health (FOPH) tracks the development of SARS-CoV-2 variants over time in Switzerland [29]. The Danish Covid-19 Genome Consortium [30] displays the number of cases with specific variants and the total number of cases sequenced each week. To calculate the proportion of each VOC, this study divided the number of cases with specific variants by the total number of cases sequenced.

Using the latest official numbers from governments and ministries of health around the world, Our World in Data [31] provides statistics on the proportion of people fully vaccinated against COVID-19. The percentage of fully vaccinated population means the percentage of a given country’s total population who have received all doses prescribed by their vaccination regimen, regardless of whether they have received booster shots. The Organization for Economic Co-operation and Development provides the latest life expectancy at birth [32] and per capita health expenditure [33] for each country.

In addition, this study used the indicators obtained by OxCGRT [21] to reflect the country’s policy changes, including policies on school and workplace closures, cancelation of public events and gatherings, stay-at-home restrictions, and international and domestic travel. The visualization of each indicator is displayed in Our World in Data [31].

### CFR Adjustment

This study used different delay times to adjust the fatality rate, namely, 1, 2, 3, and 4 weeks. The number of new deaths in the next week was divided by the number of new cases in this week to obtain the CFR adjusted with a delay time of 1 week. The rest can be deduced by analogy to obtain the CFR adjusted by 2, 3, or 4 weeks’ delay time.

### Statistical Analysis

The R Statistical Software 4.0.1 [34] and RStudio 1.4.1717 [35] were used for statistical description and analysis of all data. The R packages used in this study include “psych” [36], “betareg” [37], “mfx” [38], “broom” [39], “effects” [40], “ggplot2” [41], and “ggcorrplot” [42].

We calculated the Spearman correlation coefficient to assess the relation between different factors and delay time adjusted CFR. This study used beta regression to analyze the influencing factors. A *P*-value less than 0.05 was considered to be statistically significant in all statistical analyses in this study.

As the adjustment for CFR, the outcome variable in this study, contained a value of 0, this study performed the following transformations on the outcome variable when performing beta regression, where n is the sample size [37]:

$$y^{\prime} = \frac{{y\left( {n - 1} \right) + 0.5}}{n}$$

## Result

### Trends in CFR

**Fig. 1:**
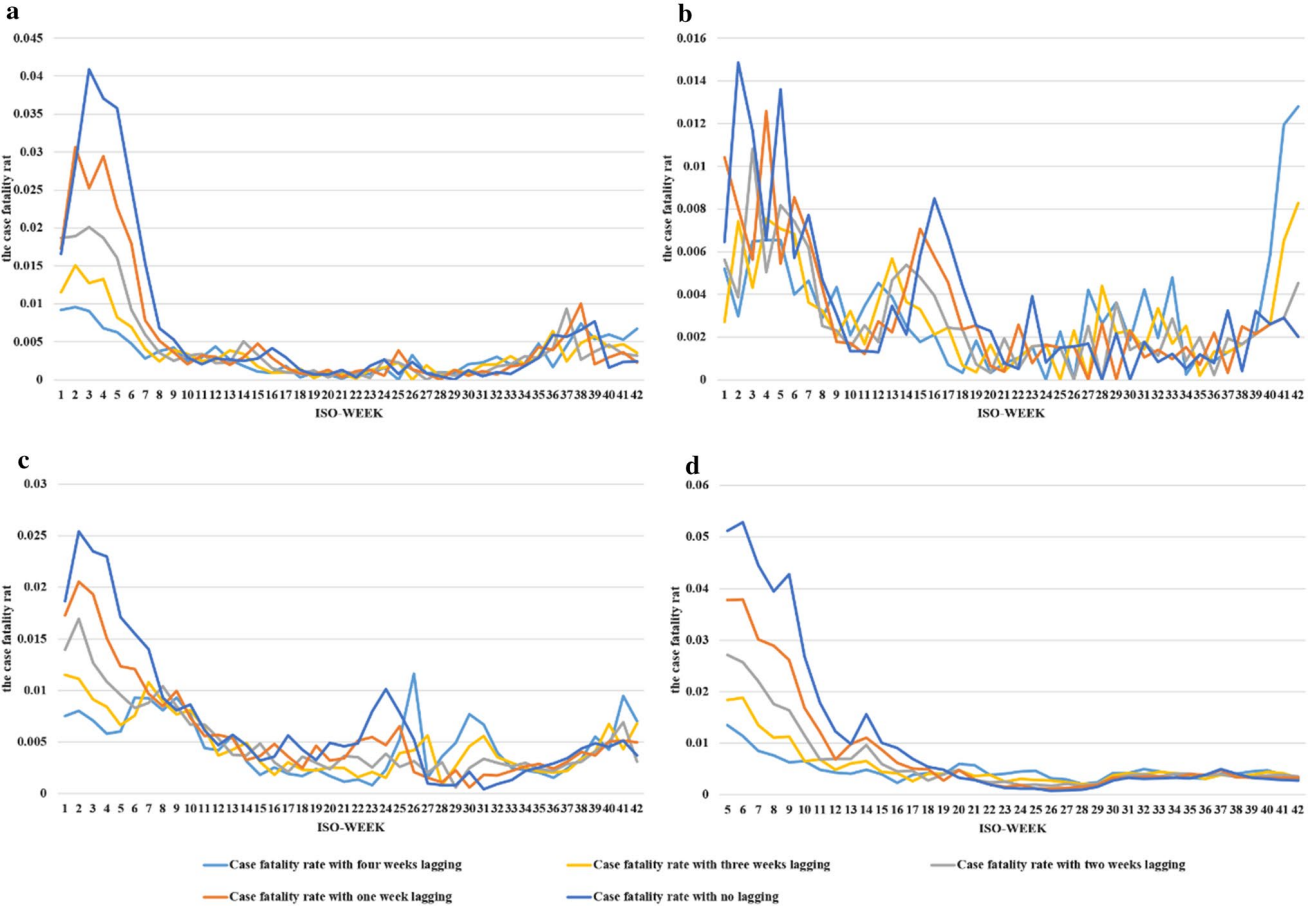
Changes in CFRs over time in the four countries. The ordinate is the CFR of the four countries with different lag times. The abscissa is the ISO-WEEK, which is the number of weeks in 2021 calculated on the basis of the ISO week date system. **a** CFR in Denmark from the 1st week to the 42nd week of 2021. **b** CFR in Norway from the 1st week to the 42nd week of 2021. **c** CFR in Switzerland from the 1st week to the 42nd week of 2021. **d** CFR in the UK from the 5th week to the 42nd week of 2021

The countries covered in this study include the UK, Denmark, Switzerland, and Norway. Using data from the WHO and national health agencies, this study calculated four adjusted CFRs with different lag times. From January 4 to October 24, 2021, the UK had the highest adjusted CFR in the 6th week of 2021. No deaths of COVID-19 cases were recorded in Denmark in week 29 and Norway in weeks 28 and 30, which contributed to their lowest adjusted CFR. The CFR of these four countries showed an overall downward trend (Fig. 1).

### Popularity of Variants

The Alpha variant has already dominated the UK from the beginning of 2021. With a steep upward trend, Alpha variants also dominate in Switzerland, Denmark, and Norway in the 6th or 7th week of 2021.

**Fig. 2:**
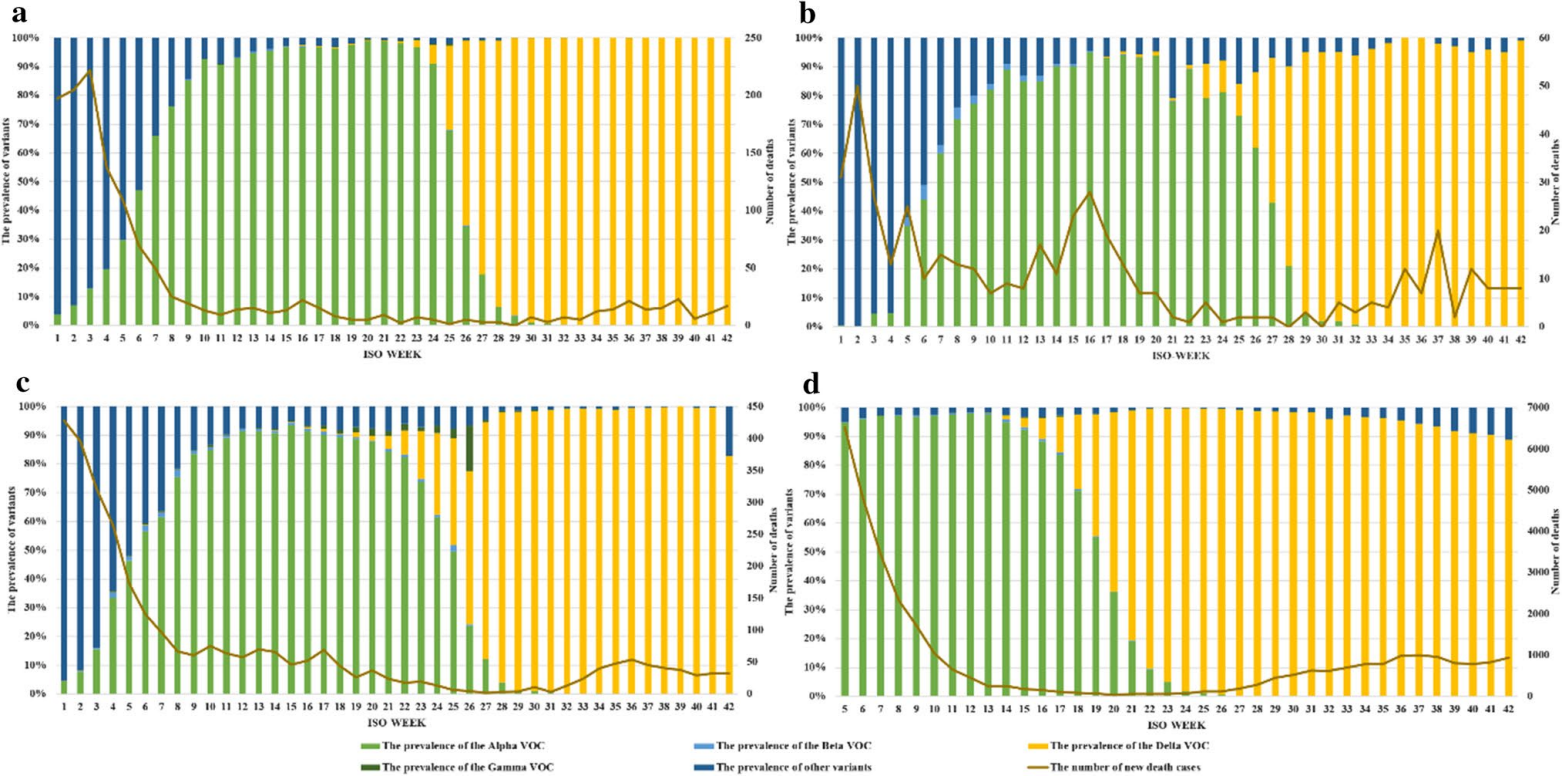
Prevalence of VOCs and the number of new death cases in four countries. The ordinate on the left is the prevalence ratio of different VOCs. The ordinate on the right is the number of patients who were hospitalized or who died. The abscissa is the ISO-WEEK, which is the same as that in Fig. 1. **a** Of Denmark from the 1st week to the 42nd week of 2021. **b** Of Norway from the 1st week to the 42nd week of 2021. **c** Of Switzerland from the 1st week to the 42nd week of 2021. **d** Of the UK from the 5th week to the 42nd week of 2021

With the spread of the Delta VOC on the European continent, the prevalence of the Delta VOC in the UK exceeded 50% in the 20th week of 2021 (Fig. 2a), which meant that it tended to be dominant. In the next few weeks, the Delta VOC gradually became the dominant variant in Switzerland (in week 26), Denmark (in week 26), and Norway (in week 27) (Fig. 2b, c, d). During this period, the Beta and Gamma VOCs were also detected, but the proportion remained below 4%. Figure 2 shows that after the Delta VOC became dominant, the number of new deaths in each week showed an increasing trend (Fig. 2).

### Proportion of Full Vaccination Against COVID-19

**Fig. 3:**
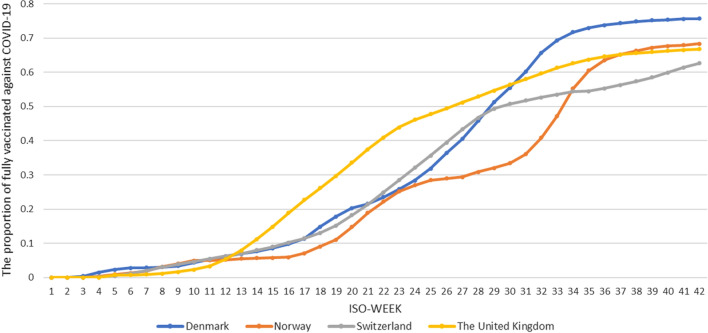
Proportion of full vaccination against COVID-19. The ordinate is the proportion of full vaccination against COVID-19 in the entire population of the given country, and the abscissa is the number of weeks in 2021 calculated on the basis of the ISO week date system

In December 2020 and January 2021, the UK, Denmark, Norway, and Switzerland had successively approved COVID-19 vaccination programs. Given that the first and second dose must be separated by at least 21 days, the proportion of full vaccination against COVID-19 in all countries in the first 4 weeks of 2021 was close to zero. Afterward, the proportion gradually increased. At the end of the study in the 42nd week of 2021, the proportion exceeded 50% in all countries. Denmark had the highest proportion of fully vaccinated people (75.71%), followed by Norway (68.32%). The percentages of fully vaccinated people in the UK and Switzerland reached 66.8% and 62.65%, respectively (Fig. 3).

### Response to the COVID-19 Pandemic

**Fig. 4:**
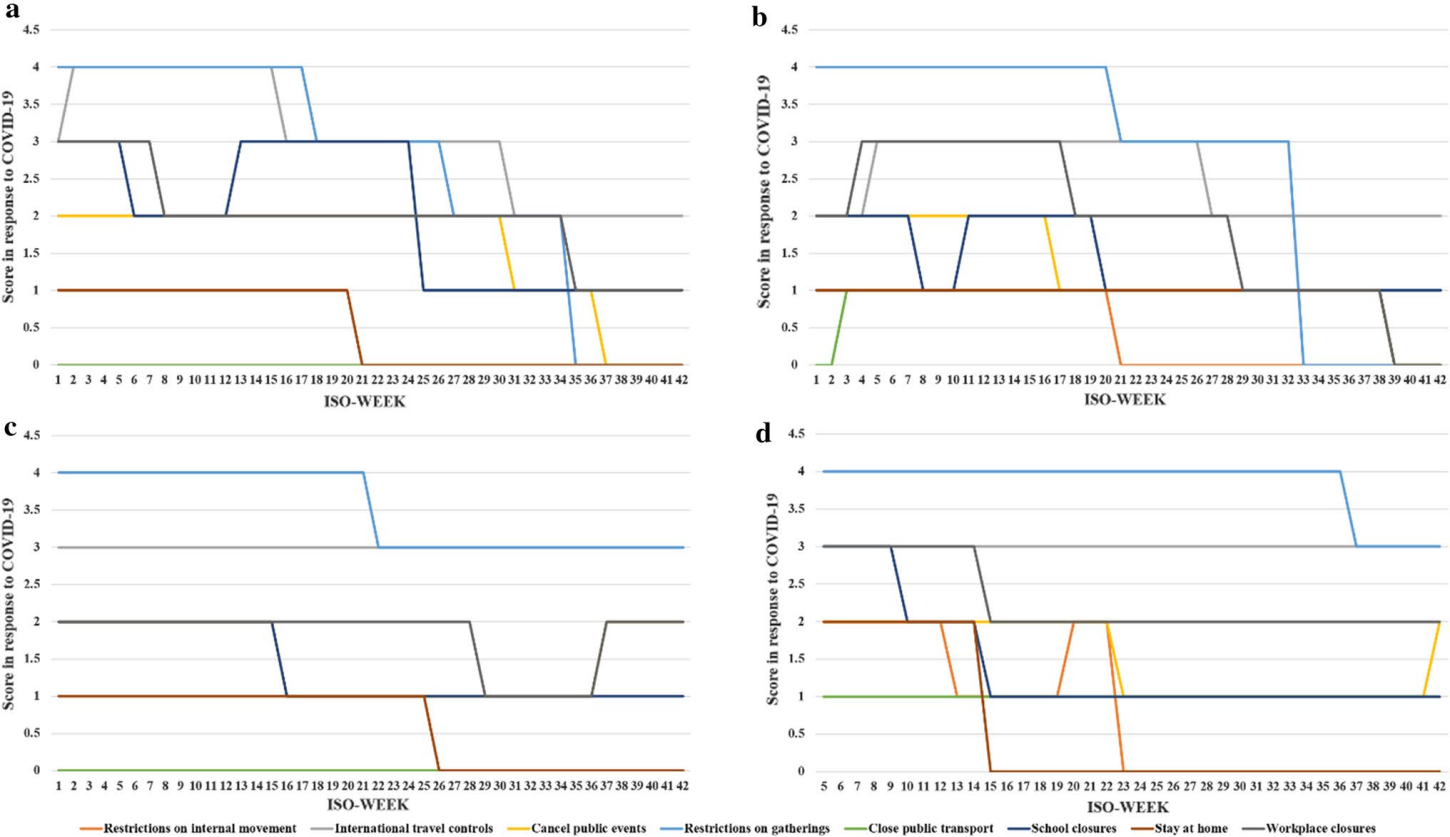
Changes in the scores in response to COVID-19. **a** Of Denmark from the 1st week to the 42nd week of 2021. **b** Of Norway from the 1st week to the 42nd week of 2021. **c** Of Switzerland from the 1st week to the 42nd week of 2021. **d** Of the UK from the 5th week to the 42nd week of 2021

This study included eight indicators to reflect the country’s response to COVID-19. In 2021, the indicator scores of these four countries were constantly changing. The scores and meanings of the indicators are shown in Appendix Table 4. The higher the score, the stricter the prevention and control measures at that time. Compared with the beginning of 2021, most of the policies became looser in the second half of 2021, and strict scores decreased (Fig. 4).

### Medical and Health Level Indicators

The indicators of the four countries included in this study are not the same. Norway had the highest life expectancy at birth (83.3), Switzerland had the highest per capita health expenditure (US$7,138.1) and the UK had the highest Global Health Security (GHS) index (83.5) (Table 1).

**Table 1:** Medical and health level indicators in four countries

Country	Life expectancy at birth	Per capita health expenditure	GHS indicators
Denmark	81.6	5849.4	70.4
Norway	83.3	6748.4	64.6
Switzerland	83.2	7138.1	67
UK	80.4	5267.7	83.5

### Correlation Analysis Results

**Fig. 5:**
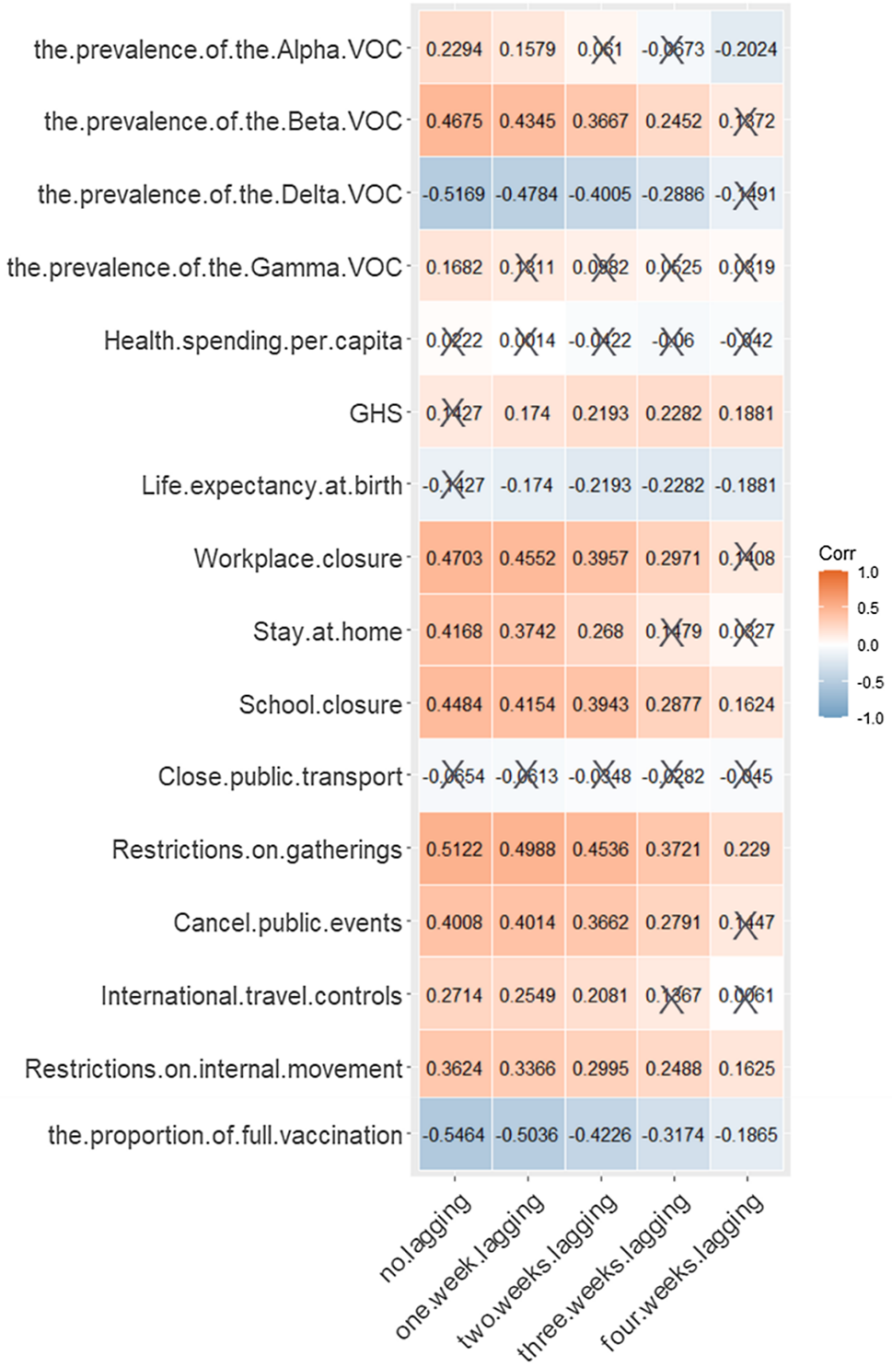
Graph of Spearman correlation coefficient matrix. The closer the color is to blue, the closer the correlation coefficient is to −1. The closer the color is to orange, the closer the correlation coefficient is to 1. The white color indicates that the correlation coefficient is 0. The black “× ” indicates that the correlation coefficient of this grid has a *P*-value greater than 0.05, which was not statistically significant

Considering the fatality rate with different lag times as the dependent variable and the popularity of variants, percentage of full vaccination, medical and health level indicators, and indicators reflecting the response to COVID-19 as independent variables, we calculated the Spearman correlation coefficient between multiple variables with statistical significance at *P* < 0.05. The detailed calculation results of the correlation are shown in Fig. 5.

### Model of Adjusted CFR with Different Lagging Time

The dependent variable of this study was the CFR adjusted for different lag times, which was a proportional variable with a value between 0 and 1. Therefore, this study used beta regression, which was more suitable for proportional variables than linear regression models to analyze influencing factors. Among the four VOCs, the Delta VOC was the main epidemic strain. Therefore, this study did not include the prevalence of the Alpha, Beta, and Gamma VOCs in beta regression.

In beta regression, a coefficient less than 0 means that the increase in this variable will reduce the fatality rate, whereas a coefficient greater than 0 means that an increase in this variable will increase the fatality rate. The farther the coefficient is from 0, the greater the degree of change in the dependent variable.

Taking the four adjusted CFRs as dependent variables separately, this study calculated the Akaike information criterion (AIC) of each model obtained by beta regression. The detailed results are shown in Table 2.

**Table 2:** Comparison of fit of different models

Dependent variable	The result of the likelihood ratio test	AIC
Case fatality with 1 week lagging	*P* < 0.001	−1496
Case fatality with 2 weeks lagging	*P* < 0.001	−1560
Case fatality with 3 weeks lagging	*P* < 0.001	−1609
Case fatality with 4 weeks lagging	*P* < 0.001	−1599

The likelihood ratio test results of the four models were all *P* < 0.001, indicating that these models were good fitting models, but the model with a lag time of 3 weeks had the lowest AIC value of -1609, which was the best among all models. Therefore, this study showed in detail the results of case fatality with 3 weeks lagging for the dependent variable. The results of the other three models are shown in the Appendix Tables 5–7.

### Results of Beta Regression on CFR with Three Weeks Lagging

The results of the beta regression of CFR lagging by 3 weeks showed that vaccination rates, restrictions on internal movement at level 1, international travel controls at level 3, cancelation of public events at levels 1 and 2, and stay at home at level 2 were statistically significant in reducing the 3 weeks lagging CFR (*P* < 0.05). School closures at level 3, workplace closures at level 3, and a higher GHS index had a statistically significant increase in 3 weeks lagging CFR (*P* < 0.05). A higher prevalence of Delta VOC also increased the fatality rate, but it was not statistically significant.

Because the logit transformation was used in the model, the coefficients of the beta regression model could not be explained directly, so this study calculated the marginal effects of each variable to explain the impact.

For every 1% increase in the percentage of full vaccination, the CFR decreased by an average of 0.66% (95% CI 0.05–1.28%). Raising the level of restrictions on internal movement from 0 to 1 was associated with an average reduction in CFR of 0.20% (95% CI 0.04–0.36%). An increase in the strictness level of international travel controls from 2 to 3 was associated with an average reduction in CFR of 0.39% (95% CI 0.20–0.58%). An increase in the severity level of cancelation of public events from 0 to 1 and 2 was associated with an average reduction in CFR of 0.49% (95% CI 0.14–0.84%) and 0.37% (95% CI 0.01–0.73%), respectively. An increase in the strictness level of stay at home from 0 to 2 was associated with an average reduction in CFR of 0.36% (0.15–0.56%). On the contrary, an increase in the severity level of school and workplace closures from 1 or 0 to 3 was associated with an average increase in CFR of 0.39% (95% CI 0.20–0.59%) and 0.83% (95% CI 0.53–1.12%), respectively. A 1-point increase in the GHS indicator score was associated with an average increase of 0.12% (95% CI 0.06–0.19%) in the CFR.

Notably, the beta regression results showed that higher prevalence of Delta VOC increases CFR, although the difference was not statistically significant. This was contrary to the correlation analysis results showing that the prevalence of Delta VOC had a significant negative correlation with CFR. Given that multivariate regression takes into account the influence of other variables, this study tended to show that the results of beta regression were more realistic and reliable.

The detailed results of the beta regression of CFR lagging by 3 weeks are shown in Table 3.

**Table 3:** The detailed results of the beta regression of the case fatality rate lagging by three weeks

Dependent Variable	Independent Variable	Coefficients(95%CI)	*P*^b^	Margins(95%CI)
Fatality-3 weeks lagging	(intercept)	−81.297 (−181.597, 19.002)	0.112	–
	the prevalence of the Delta VOC	0.157 (−0.253, 0.568)	0.453	0.0011 (−0.0018, 0.0040)
	the percentage of full vaccination	−0.933 (−1.790, −0.076)	***0.033***	−0.0066 (−0.0128, −0.0005)
	Restrictions on internal movement			
	0	Reference		
	1	−0.301 (−0.557, −0.046)	***0.021***	−0.0020 (−0.0036, −0.0004)
	2	−0.202 (−0.472, 0.069)	0.144	−0.0014 (−0.0032, 0.0004)
	International travel controls^a^			
	2	Reference		
	3	−0.476 (−0.673, −0.278)	** < *0.001***	−0.0039 (−0.0058, −0.0020)
	4	−0.128 (−0.409, 0.154)	0.373	−0.0012 (−0.0039, 0.0015)
	Cancel public events			
	0	Reference		
	1	−0.582 (−0.903, −0.261)	** < *0.001***	−0.0049 (−0.0084, −0.0014)
	2	−0.410 (−0.746, −0.073)	*0.017*	−0.0037 (−0.0073, −0.0001)
	Restrictions on gatherings^a^			
	0	Reference		
	2	0.141 (−0.267, −0.549)	0.497	0.0013 (−0.0025, 0.0052)
	3	−0.228 (−0.547, −0.091)	0.160	−0.0018 (−0.0046, 0.0010)
	4	−0.250 (−0.668, −0.167)	0.240	
	Close public transport^a^			
	0	Reference		
	1	−0.193 (−0.554, −0.168)	0.295	−0.0014 (−0.0039, 0.0012)
	School closures^a^			
	1	Reference		
	2	0.155 (−0.010, −0.321)	0.066	0.0011 (−0.0001, 0.0022)
	3	0.484 (0.270, −0.698)	** < *0.001***	0.0039 (0.0020, 0.0059)
	Stay at home^a^			
	0	Reference		
	1	−0.199 (−0.456, 0.058)	0.129	−0.0015 (−0.0035, 0.0005)
	2	−0.548 (−0.881, −0.215)	***0.001***	−0.0036 (−0.0056, −0.0015)
	Workplace closures			
	0	Reference		
	1	0.543 (−0.004.1.090)	0.052	0.0026 (0.0005, −0.0048)
	2	0.558 (−0.001, 1.116)	0.050	0.0027 (0.0006, −0.0049)
	3	1.184 (0.589, 1.788)	** < *0.001***	0.0083 (0.0053, −0.0112)
	Life expectancy at birth	0.771 (−0.467, −2.008)	0.222	0.0055 (−0.0033, −0.0143)
	Health spending per capita	0.0002 (−0.001, −0.002)	0.722	0.0000 (0.0000, −0.0000)
	GHS	0.173 (0.080, −0.265)	** < *0.001***	0.0012 (0.0006, −0.0019)

^a^Because the countries included in this study did not cover all levels in their response to COVID-19, not all levels were covered in the regression

^b^*P*-values for regression model coefficients

Italics and bold indicated statistical significance

## Discussion

After the emergence and dominance of VOCs, more and more studies have focused on the changes in the virulence of VOCs. Most of them were cohort studies or case–control studies, and only a small part of them involved ecological research, including two studies on the Gamma VOC (variant P.1) in Amazonia, Brazil [10, 43]. This study adopted the design of ecological research to explore the factors that may affect the population CFR.

A study in Australians found that the estimated average time from diagnosis to death of COVID-19 patients was 18.1 days [44]. This finding supported the adjustment of the CFR in this study. We calculated the adjusted CFR with different lag times, which reduced the error in fatality estimation caused by the time difference between death and diagnosis, and improved the reliability of this research.

When the CFR adjusted time was 2 and 3 weeks, the regression results showed that a high proportion of Delta variants was associated with a higher CFR, but it was not statistically significant. At a case-fatality-adjusted time of 4 weeks, the regression results indicated that a high proportion of the Delta variant was statistically significantly associated with a high case fatality rate. The presence of VOCs across the time window covered by this study included Alpha, Beta, Gamma, and Delta, suggesting that the lethality of Delta VOC was higher than that of the other three VOCs. This is similar to the results of other studies, which found that patients infected with Delta VOC had a higher risk of hospitalization or even death [45, 46]. Countries where Delta VOC was the dominant strain need to make more efforts to reduce the CFR.

The results of this study found that the higher percentage of full vaccination was the factor leading to decreased CFR. This was consistent with the results of previous studies. A study [47] in the US found that unvaccinated people were 10 times more likely to die from COVID-19 than those who had completed the vaccination. These results all proved that the current strategy of widespread COVID-19 vaccination worldwide was beneficial and effective.

In the regression analysis, the results showed that stricter measures on restrictions on internal movement, international travel controls, and cancelation of public events may reduce the CFR. Other studies also found that policy adjustments will affect the fatality rate [20], and prevention and control policies will affect the cumulative number of confirmed cases [48]. These results all showed that strictly reducing aggregation was effective to control the spread and prevalence of infectious diseases. However, the regression results of this study also showed that strict measures on school and workplace closures were factors that may increase the CFR. Unlike measures that restrict gatherings, school or workplace closures may lead to more free time for people, which may increase their movement. This is similar to the findings of a past study that closing schools would increase the total number of deaths from COVID-19 [49]. This may be because children and adolescents will have increased contact with other household members following school closures and may not strictly follow other precautions needed to stop transmission. Moreover, this study was unable to clarify the causal relationship between strict closure measures and higher fatality rates. As a macro-level prevention and control method, more research is needed to ascertain the impact of national policies.

This study used three indicators to reflect the country’s sanitation and health level, including life expectancy at birth, health spending per capita, and GHS. The regression results showed higher GHS index was a factor that increased CFR. Similarly, a study indicated that the GHS index was positively associated with total deaths per million, but the GHS index cannot perfectly assess a country’s ability to respond to a global pandemic [50]. Aitken et al. also found a positive correlation between the GHS index and detection rate, and that countries with a high GHS index also had a large burden of COVID-19 [51]. Countries with high GHS indices have stronger health systems and may be able to more adequately identify deaths during the COVID-19 pandemic, which may partly explain why higher GHS indices are associated with higher case fatality rates.

The advantage of this study is that a newer and more appropriate method was used in the statistical analysis, which provided evidence for the reliability of the results. When there are many candidate variables, as in this study, choosing an appropriate model has a significant impact on the results. In linear regression, the variables included in the model are usually selected on the basis of the *P*-value of univariate analysis. Researchers are often unable to determine the specific meaning of the excluded variables. Given that the distribution of proportional data is often skewed, deviations may occur due to data conversion when using general linear regression analysis. Moreover, the results predicted by the regression model may exceed the range of [0, 1], resulting in insufficient practical significance of the model. At present, various analysis methods are more suitable for proportional data [52], such as beta regression [53] and Dirichlet regression [54]. The present study used beta regression when analyzing the influencing factors and establishing the model. The results will be more reliable than using traditional linear regression.

This research still had shortcomings. The area covered by ecological research was not wide enough, and the results obtained could not be extrapolated to all countries in the world unconditionally. In addition, the data used in this study were all second-hand data, which might lead to information bias. Nevertheless, the results of this study could still provide insights into predicting the fatality rate and adjusting prevention and control measures.

## Conclusion

This ecological study showed that the higher prevalence of the Delta VOC, stricter measures on school and workplace closures, and higher GHS index were factors that might cause an increase in the adjusted fatality rate. The higher percentage of full vaccination and higher levels of restrictions on internal movement, international travel controls, cancelation of public events, and stay-at-home restrictions were factors that might cause a decrease in the adjusted fatality rate.

## Data Availability

All data were obtained at the request of the corresponding author.

## Appendix

### Scores and Meanings of the Indicators

See Table 4.

**Table 4:** The scores levels and meanings of the indicators

Indicators	Level	Meaning
Restrictions on internal movement	0	No measures
	1	Recommend movement restriction
	2	Restrict movement
International travel controls	0	No measures
	1	Screening
	2	Quarantine arrivals from high-risk regions
	3	Ban on high-risk regions
	4	Total border closure
Cancel public events	0	No measures
	1	Recommend cancelling
	2	Require cancelling
Restrictions on gatherings	0	No restrictions
	1	Restrictions on very large gatherings (the limit is above 1,000 people)
	2	Restrictions on gatherings between 100 and 1,000 people
	3	Restrictions on gatherings between 10 and 100 people
	4	Restrictions on gatherings of less than 10 people
Close public transport	0	No measures
	1	Recommend closing (or significantly reduce volume/route/means of transport available)
	2	Require closing (or prohibit most citizens from using it)
School closures	0	No measures
	1	recommend closing
	2	Require closing (only some levels or categories, e.g., just high school, or just public schools)
	3	Require closing all levels
Stay at home	0	No measures
	1	recommend not leaving house
	2	require not leaving house with exceptions for daily exercise, grocery shopping, and ‘essential’ trips
	3	Require not leaving house with minimal exceptions (e.g., allowed to leave only once every few days, or only one person can leave at a time, etc.)
Workplace closures	1	recommend closing (or work from home)
	2	require closing (or work from home) for some sectors or categories of workers
	3	require closing (or work from home) all but essential workplaces (e.g., grocery stores, doctors)

### Results of Beta Regression on CFR with One Week Lagging

The result of beta regression showed that for every 1% increase in the percentage of full vaccination, the CFR decreases by an average of 1.37% (95% CI 0.39–2.36%). Raising the level of restrictions on internal movement from 0 to 1 was associated with an average reduction in CFR of 0.30% (95% CI 0.05–0.55%). An increase in the strictness level of international travel controls from 2 to 3 was associated with an average reduction in CFR of 0.66% (95% CI 0.32–1.00%). An increase in the severity level of cancelation of public events from 0 to 1 and 2 was associated with an average reduction in CFR of 0.83% (95% CI 0.17–1.48%) and 0.70% (95% CI 0.02–1.38%), respectively. An increase in the strictness level of restrictions on gatherings from 0 to 3 and 4 was associated with an average reduction in CFR of 0.80% (95% CI 0.01–1.60%) and 1.02% (95% CI 0.10–1.94%), respectively. An increase in the strictness level of closure of public transport from 0 to 1 was associated with an average reduction in CFR of 0.64% (95% CI 0.23–1.06%). An increase in the strictness level of stay-at-home restriction from 0 to 2 was associated with an average reduction in CFR of 0.54% (95% CI 0.24–0.85%). On the contrary, an increase in the severity level of school closures from 1 to 3 was associated with an average increase in CFR of 0.64% (95% CI 0.35–0.94%). An increase in the severity level of workplace closures from 0 to 1, 2, and 3 was associated with an average increase in CFR of 0.48% (95% CI 0.29–0.67%), 0.56% (95% CI 0.40–0.71%), and 1.80% (95% CI 1.40–2.19%), respectively. Each additional year of life expectancy at birth was associated with an average increase of 1.82% (95% CI 0.55–3.09%) in the CFR. A 1-point increase in the GHS indicator score was associated with an average increase of 0.29% (95% CI 0.19–0.38%) in the CFR.

The detailed results of the beta regression of CFR lagging by 1 week are shown in Table 5.

**Table 5:** The detailed results of the beta regression of the case fatality rate lagging by 1 week

Dependent variable	Independent variable	Coefficients (95%CI)	*P* ^b^	Margins (95%CI)
Fatality-1 week lagging	(Intercept)	−197.716 (−318.393, −77.039)	***0.001***	–
	The prevalence of the Delta VOC	−0.0355 (−0.576, 0.505)	0.898	−0.0003 (−0.0049, 0.0043)
	The percentage of full vaccination	−1.603 (−2.75, −0.458)	***0.006***	−0.0137 (−0.0236, −0.0039)
	Restrictions on internal movement			
	0	Reference		
	1	−0.375 (−0.703, −0.047)	***0.025***	−0.0030 (−0.0055, −0.0005)
	2	−0.252 (−0.602, 0.097)	0.157	−0.0021 (−0.0050, 0.0007)
	International travel controls^a^			
	2	Reference		
	3	−0.637 (−0.896, −0.378)	** < ** ***0.001***	−0.0066 (−0.0100, −0.0032)
	4	−0.084 (−0.440, 0.272)	0.645	−0.0011 (−0.0059, 0.0036)
	Cancel public events			
	0	Reference		
	1	−0.749 (−1.18, −0.318)	** < ** ***0.001***	−0.0083 (−0.0148, −0.0017)
	2	−0.593 (−1.04, −0.142)	***0.010***	−0.0070 (−0.0138, −0.0002)
	Restrictions on gatherings^a^			
	0	Reference		
	2	0.045 (−0.515, 0.604)	0.876	0.0008 (−0.0092, 0.0108)
	3	−0.605 (−1.038, −0.174)	***0.006***	−0.0080 (−0.0160, −0.0001)
	4	−0.852 (−1.409, −0.295)	***0.003***	−0.0102 (−0.0194, −0.0010)
	Close public transport^a^			
	0	Reference		
	1	−0.721 (−1.138, −0.304)	** < ** ***0.001***	−0.0064 (−0.0106, −0.0023)
	School closures^a^			
	1	Reference		
	2	0.090 (−0.122, 0.302)	0.403	0.0007 (−0.0009, 0.0023)
	3	0.634 (0.371, 0.897)	** < ** ***0.001***	0.0064 (0.0035, 0.0094)
	Stay at home^a^			
	0	Reference		
	1	−0.104 (−0.427, 0.159)	0.528	−0.0010 (−0.0043, 0.0022)
	2	−0.723 (−1.132, −0.313)	** < ** ***0.001***	−0.0054 (−0.0085, −0.0024)
	Workplace closures			
	0	Reference		
	1	1.378 (0.678, 2.078)	** < ** ***0.001***	0.0048 (0.0029, 0.0067)
	2	1.491 (0.772, 2.210)	** < ** ***0.001***	0.0056 (0.0040, 0.0071)
	3	2.506 (1.742, 3.270)	** < ** ***0.001***	0.0180 (0.0140, 0.0219)
	Life expectancy at birth	2.123 (0.635, 3.611)	***0.005***	0.0182 (0.0055, 0.0309)
	Health spending per capita	−0.0007 (−0.0009, 0.002)	0.351	−0.0000 (−0.0000, 0.0000)
	GHS	0.337 (0.225, 0.449)	** < ** ***0.001***	0.0029 (0.0019, 0.0038)

^a^Because the countries included in this study did not cover all levels in their response to COVID-19, not all levels were covered in the regression

^b^*P*-values for regression model coefficients

Italics and bold indicated statistical significance

### Results of Beta Regression on CFR with Two Weeks Lagging

For every 1% increase in the percentage of full vaccination, the CFR decreases by an average of 1.02% (95% CI 0.27–1.77%). Raising the level of restrictions on internal movement from 0 to 1 was associated with an average reduction in CFR of 0.26% (95% CI 0.07–0.45%). An increase in the strictness level of international travel controls from 2 to 3 was associated with an average reduction in CFR of 0.42% (95% CI 0.18–0.66%). An increase in the severity level of cancelation of public events from 0 to 1 and 2 was associated with an average reduction in CFR of 0.71% (95% CI 0.22–1.20%) and 0.59% (95% CI 0.09–1.10%), respectively. An increase in the strictness level of stay at home from 0 to 2 was associated with an average reduction in CFR of 0.40% (95% CI 0.15–0.64%). On the contrary, an increase in the severity level of school closures from 1 to 3 was associated with an average increase in CFR of 0.59% (95% CI 0.35–0.84%). An increase in the severity level of workplace closures from 0 to 1, 2, and 3 was associated with an average increase in CFR of 0.37% (95% CI 0.17–0.57%), 0.41% (95% CI 0.22–0.60%), and 1.15% (95% CI 0.85–1.46%), respectively. A 1-point increase in the GHS indicator score was associated with an average increase of 0.16 (95% CI 0.08–0.24%) in the CFR.

The detailed results of the beta regression of CFR lagging by 2 weeks are shown in Table 6.

**Table 6:** The detailed results of the beta regression of the case fatality rate lagging by 2 weeks

Dependent Variable	Independent Variable	Coefficients (95%CI)	*P* ^b^	Margins (95%CI)
Fatality-2 weeks lagging	(Intercept)	−98.908 (−212.7, 14.86)	0.088	–
	The prevalence of the Delta VOC	0.133 (−0.332, 0.598)	0.574	0.0010 (−0.0025, 0.0046)
	The percentage of full vaccination	−1.335 (−2.313, −0.356)	***0.008***	−0.0102 (−0.0177, −0.0027)
	Restrictions on internal movement			
	0	Reference		
	1	−0.362 (−0.647. −0.077)	***0.013***	−0.0026 − (-0.0045, −0.0007)
	2	−0.289 (−0.595, 0.017)	0.064	−0.0021 (−0.0043, −0.0000)
	International travel controls^a^			
	2	Reference		
	3	−0.478 (−0.703, −0.252)	** < ** ***0.001***	−0.0042 (−0.0066, −0.0018)
	4	−0.054 (−0.366, 0.258)	0.735	−0.0006 (−0.0039, 0.0028)
	Cancel public events			
	0	Reference		
	1	−0.722 (−1.091, −0.354)	** < ** ***0.001***	−0.0071 (−0.0120, −0.0022)
	2	−0.565 (−0.949, −0.180)	***0.004***	−0.0059 (−0.0110, −0.0009)
	Restrictions on gatherings^a^			
	0	Reference		
	2	0.071 (−0.403, 0.545)	0.769	0.0009 −0.0051, 0.0068)
	3	−0.450 (−0.817, −0.083)	***0.016***	−0.0044 (−0.0090, 0.0002)
	4	−0.547 (−1.021, −0.073)	***0.024***	−0.0052 (−0.0106, 0.0003)
	Close public transport^a^			
	0	Reference		
	1	−0.230 (−0.640, 0.180)	0.271	−0.0018 (−0.0049, 0.0014)
	School closures^a^			
	1	Reference		
	2	0.168 (−0.0174, 0.353)	0.076	0.0012 (−0.0001, 0.0025)
	3	0.651 (0.416, 0.886)	** < ** ***0.001***	0.0059 (0.0035, 0.0084)
	Stay at home^a^			
	0	Reference		
	1	−0.167 (−0.450, 0.116)	0.247	−0.0014 (−0.0038, 0.0010)
	2	−0.569 (−0.933, −0.205)	***0.002***	−0.0040 (−0.0064, −0.0015)
	Workplace closures			
	0	Reference		
	1	0.877 (0.249, 1.504)	***0.006***	0.0037 (0.0017, 0.0057)
	2	0.941 (0.298, 1.584)	***0.004***	0.0041 (0.0022, 0.0060)
	3	1.696 (1.017, 2.375)	** < ** ***0.001***	0.0115 (0.0085, 0.0146)
	Life expectancy at birth	0.953 (−0.451, 2.357)	0.183	0.0073 (−0.0034, 0.0180)
	Health spending per capita	0.0002 (−0.001, 0.002)	0.752	0.0000 (−0.000, 0.0000)
	GHS	0.211 (0.106, 0.316)	** < ** ***0.001***	0.0016 (0.0008, 0.0024)

^a^Because the countries included in this study did not cover all levels in their response to COVID-19, not all levels were covered in the regression

^b^*P*-values for regression model coefficients

Italics and bold indicated statistical significance

### Results of Beta Regression on CFR with Four Weeks Lagging

For every 1% increase in the prevalence of the Delta VOC, the CFR increased by an average of 0.31% (95% CI 0.01–0.60%). For every 1% increase in the percentage of full vaccination, the CFR decreased by an average of 0.87% (95% CI 0.26–1.48%). An increase in the strictness level of international travel controls from 2 to 3 was associated with an average reduction in CFR of 0.38% (95% CI 0.19–0.57%). An increase in the severity level of cancelation of public events from 0 to 1 and 2 was associated with an average reduction in CFR of 0.67% (95% CI 0.27–1.07%) and 0.57% (95% CI 0.16–0.98%), respectively. An increase in the strictness level of stay at home from 0 to 2 was associated with an average reduction in CFR of 0.34% (95% CI 0.12–0.55%). On the contrary, an increase in the severity level of school closures from 1 to 3 was associated with an average increase in CFR of 0.21% (95% CI 0.03–0.39%). An increase in the severity level of workplace closures from 0 to 3 was associated with an average increase in CFR of 0.49% (95% CI 0.13–0.85%).

The detailed results of the beta regression of CFR lagging by 4 weeks are shown in Table 7.

**Table 7:** The detailed results of the beta regression of the case fatality rate lagging by 4 weeks

Dependent Variable	Independent Variable	Coefficients (95%CI)	*P* ^b^	Margins(95%CI)
Fatality-4 weeks lagging	(intercept)	−14.566 (−120.104, 90.073)	0.787	–
	the prevalence of the Delta VOC	0.438 (0.019, 0.857)	***0.040***	0.0031 (0.0001, 0.0060)
	the percentage of full vaccination	−0.250 (−2.123, −0.376)	***0.005***	−0.0087 (−0.0148, −0.0026)
	Restrictions on internal movement			
	0	Reference		
	1	−0.221 (−0.484, 0.043)	0.101	−0.0014 (−0.0031, 0.0002)
	2	−0.096 (−0.370, 0.179)	0.494	−0.0007 (−0.0025, 0.0012)
	International travel controls^a^			
	2	Reference		
	3	−0.476 (−0.678, −0.275)	** < ** ***0.001***	−0.0038 (−0.0057, −0.0019)
	4	−0.201 (−0.501, 0.089)	0.171	−0.0019 (−0.0045, 0.0008)
	Cancel public events			
	0	Reference		
	1	−0.744 (−1.066, −0.421)	** < ** ***0.001***	−0.0067 (−0.0107, −0.0027)
	2	−0.591 (−0.929, −0.253)	** < ** ***0.001***	−0.0057 (−0.0098, −0.0016)
	Restrictions on gatherings^a^			
	0	Reference		
	2	−0.012 (−0.424, 0.399)	0.953	−0.0001 (−0.0033, 0.0031)
	3	−0.082 (−0.406, 0.242)	0.618	−0.0006 (−0.0031, 0.0019)
	4	−0.140 (−0.567, 0.288)	0.522	−0.0010 (−0.0042, 0.0022)
	Close public transport^a^			
	0	Reference		
	1	0.048 (−0.335, 0.432)	0.805	0.0003 (−0.0024, 0.0030)
	School closures^a^			
	1	Reference		
	2	0.149 (−0.024, 0.321)	0.091	0.0010 (−0.0002, 0.0023)
	3	0.280 (0.054, 0.506)	***0.015***	0.0021 (0.0003, 0.0039)
	Stay at home^a^			
	0	Reference		
	1	−0.238 (−0.507, 0.031)	0.083	−0.0018 (−0.0038, 0.0003)
	2	−0.519 (−0.873, −0.165)	***0.004***	−0.0034 (−0.0055, −0.0012)
	Workplace closures			
	0	Reference		
	1	0.134 (−0.424, 0.692)	0.638	0.0007 (−0.0022, 0.0037)
	2	0.248 (−0.321, 0.817)	0.392	0.0015 (−0.0015, 0.0045)
	3	0.667 (0.059, 1.274)	***0.032***	0.0049 (0.0013, 0.0085)
	Life expectancy at birth	−0.011 (−1.313, 1.291)	0.987	−0.0001 (−0.0091, 0.0090)
	Health spending per capita	0.0008 (−0.0005, 0.002)	0.230	0.0000 (−0.0000, 0.0000)
	GHS	0.090 (−0.007, 0.187)	0.071	0.0006 (−0.0001, 0.0013)

^a^Because the countries included in this study did not cover all levels in their response to COVID-19, not all levels were covered in the regression

^b^*P*-values for regression model coefficients

Italics and bold indicated statistical significance

## Abbreviations

CFR: Case fatality rate
GHS: The Global Health Security
WHO: The World Health Organization
COVID-19: Coronavirus disease 2019
SARS-CoV-2: Severe acute respiratory syndrome coronavirus-2
VOCs: Variants of concern
OxCGRT: The oxford Covid-19 government response tracker
ISO: The International Organization for Standardization
FOPH: The Swiss Federal Office of Public Health
AIC: The Akaike information criterion
